# Assessment of Aflatoxin and Fumonisin Contamination and Associated Risk Factors in Feed and Feed Ingredients in Rwanda

**DOI:** 10.3390/toxins11050270

**Published:** 2019-05-14

**Authors:** Kizito Nishimwe, Erin Bowers, Jean de Dieu Ayabagabo, Richard Habimana, Samuel Mutiga, Dirk Maier

**Affiliations:** 1 Department of Food Science and Human Nutrition, Iowa State University, Ames, IA 50011, USA; 2 School of Agriculture and Food Science, University of Rwanda, PO Box 4285 Kigali, Rwanda; 3 Department of Agricultural and Biosystems Engineering, Iowa State University, Ames, IA 50011, USA; erin@iastate.edu (E.B.); dmaier@iastate.edu (D.M.); 4 School of Animal Sciences and Veterinary Medicine, University of Rwanda, PO Box 4285 Kigali, Rwanda; ayabagabojean87@yahoo.fr (J.d.D.A.); hrichard86@yahoo.fr (R.H.); 5 Biosciences Eastern and Central Africa and International Livestock Research Institute (BecA ILRI) Hub, ILRI Complex, Along Old Naivasha Road, PO Box 30709–GPO 00100 Nairobi, Kenya; s.mutiga@cgiar.org

**Keywords:** aflatoxin, fumonisin, animal feed safety, dairy farmers, poultry farmers, Rwanda

## Abstract

Mycotoxins are fungal metabolites that contaminate crops, food, and animal feeds. Aflatoxins and fumonisins are among the mycotoxins that have been increasingly reported to affect health and productivity of livestock globally. Given that the health and productivity of livestock can directly influence human food safety and security, a study was conducted to assess the levels and factors for aflatoxin and fumonisin contamination in feed and feed ingredients in Rwanda. Aflatoxins and fumonisins were analyzed in 3328 feed and feed ingredient samples collected at six time points between March and October 2017 in all 30 districts of Rwanda. Of the 612 participants providing samples, there were 10 feed processors, 68 feed vendors, 225 dairy farmers, and 309 poultry farmers. Enzyme-Linked Immunosorbent Assay (ELISA) was used for aflatoxin and fumonisin analyses. Mean aflatoxin levels of 108.83 µg/kg (Median (MD): 43.65 µg/kg), 103.81µg/kg (MD: 48.4 µg/kg), 88.64 µg/kg (MD: 30.90 µg/kg), and 94.95 µg/kg (MD: 70.45 µg/kg) were determined for dairy farmers, poultry farmers, feed vendors, and feed processors, respectively. Mean fumonisin levels were 1.52 mg/kg (MD: 0.71 mg/kg), 1.21 mg/kg (MD: 0.56 mg/kg), 1.48 mg/kg (MD: 0.76 mg/kg), and 1.03 mg/kg (MD: 0.47 mg/kg) for dairy farmers, poultry farmers, feed vendors, and feed processors, respectively. Aflatoxin contamination was significantly affected by time of sampling and district from which feed samples originated (*p* < 0.05). Fumonisins did not show any correlation trends. Ninety-two percent of survey participants were unaware of aflatoxins and fumonisins and their adverse effects. This study has provided the basic understanding of the extent of feed contamination across the country and has established a baseline for future interventions in Rwanda. Further studies are needed to explore strategies for mitigating mycotoxins in the feed value chain in Rwanda.

## 1. Introduction

Mycotoxins are secondary metabolites produced by some fungi under specific, favorable climate conditions. Aflatoxins and fumonisins are two classes of mycotoxins with widespread prevalence in cereal crops and feeds [1,2,3,4,5]. Aflatoxins are a group of structurally similar compounds produced by *Aspergillus* fungal species, mainly *Aspergillus flavus* and *A. parasiticus* [6]. Analyses for total aflatoxins typically include the sum of four principal aflatoxins (B1, B2, G1, and G2). Fumonisins are predominantly produced by *Fusarium verticillioides* and *F. proliferatum* [7]. Analyses for total fumonisins typically include fumonisin B1, B2, and B3. The optimum growth of *A. flavus* and *A. parasiticus* occur over a temperature range of 29–37 °C, with water activity (a_w_) of 0.99 [8]. *F. proliferatum* and *F. verticillioides* produce fumonisins on maize grain at 25–30 °C with maximum produced at 0.956 and 0.968 a_w_ [9].

Aflatoxins and fumonisins are a concern for public and animal health worldwide. Human health effects of fumonisins include disruption of sphingolipid metabolism and inhibition of folate transport, which can result in fetal neural tube defects [10]. Epidemiological studies suggest dietary fumonisin consumption may be associated with esophageal cancer [11,12,13]. Noteworthy detrimental health effects in animals resulting from fumonisin exposure include Equine Leukoencephalomalacia (ELEM) and Porcine Pulmonary Edema (PPE) [14]. Aflatoxins are classified by the International Agency for Research on Cancer (IARC) in Group 1 as carcinogenic to humans and animals. They are associated with immunosuppression [15,16,17] and childhood stunting [18,19,20] and are lethal in high doses. An outbreak in Kenya in 2004 resulted in 317 acute poisonings and 125 human deaths attributable to aflatoxins [21,22,23].

In Africa, mycotoxin contamination of commodities and animal feeds poses significant risk to the health and productivity of livestock consuming affected feed. Additionally, it poses a risk to humans that consume affected grain and animal source foods (ASF, i.e., meat, milk, and eggs) produced from animals fed mycotoxin-contaminated feed. Studies conducted in Eastern Africa revealed a high number of feed samples contaminated with aflatoxins in Kenya [24,25,26], Uganda [27], and Tanzania [28]. Livestock that consume aflatoxin-contaminated feed experience reduced productivity and detrimental health effects. A dairy herd exposed to contaminated feed (120 μg/kg of aflatoxins) for several months showed severe health problems and a decrease in milk production up to 28%. Up to 6% of dietary AFB1 transfers to milk in the form of AFM1 when lactating mammals (e.g., cows) are fed AFB1-contaminated feed [29,30]. Aflatoxin M1 (AFM1) is a metabolite of aflatoxin B1 (AFB1) that is excreted in urine, bile, feces, and milk after dietary exposure of dairy cattle to contaminated feed [31]. In chicken, aflatoxins reduce body weight, feed conversion efficiency, average daily gain, feed conversion ratios, and egg production [32]. This results in additional potential for human dietary exposure. Aflatoxin and fumonisin feed contamination is a challenge throughout Africa. It impacts livestock health and productivity, and food and feed safety [33].

Aflatoxins also have negative economic impacts. In countries like the United States, financial impacts in the form of yield loss and product downgrading, discounting, and rejection to the maize value chain due to aflatoxin contamination occur. Losses have been estimated to exceed $1 billion during years with warm summers and drought conditions based on models for estimating the potential market loss to the maize industry from aflatoxin contamination [34]. In emerging economies, aflatoxins reduce farmer incomes due to low market value of contaminated products, production rejection, and exclusion from high-value markets [35,36].

The livestock sector in Rwanda continues to grow. The percentage of livestock-owning households rearing cattle has increased from 34.4% in 2005/06 to 50.4% in 2013/14. The value of milk exports was $13,061,738 in 2016/17, a twofold increase compared to 2011/12 according to the National Institute of Statistics of Rwanda [37]. The value of live poultry export exceeded $5 million in 2015/2016 [37]. The government is contributing to growth in the livestock and poultry sectors through financial and technical assistance programs. Government efforts to increase dietary diversity and enhance human nutrition through consumption of ASF are evidenced in the countrywide Girinka (literally translated “One Cow per Poor Family”) Program [38]. There is a high demand for feeds, feed supplements, and concentrates to support the increasing production; however, the feed sector faces challenges because of mycotoxins. A limited collection of 20 feed samples from feed vendors showed evidence of high aflatoxin contamination [39]. A study conducted in Kenya has shown that maize bran is the most contaminated of all ingredients used in feedstuffs [28]. This is concerning because maize bran was reported to be the main basal feedstuff used in dairy cattle and poultry rations [40,41].

There is a scarcity of data regarding grain and feed contamination with aflatoxins and fumonisins in Rwanda that hampers mycotoxin management and mitigation efforts. The lack of data undermines government efforts to increase human health through increased consumption of ASF because they are likely also contaminated. Dietary diversity initiatives are ineffective when toxic contaminants, like aflatoxins, are present which are harmful to human health. This study was undertaken to assess the prevalence of aflatoxins and fumonisins in animal feeds and feed ingredients in Rwanda and to better understand risk factors contributing to mycotoxin contamination in animal feed.

## 2. Results

### 2.1. Aflatoxin and Fumonisin Contamination among Participants

A total of 3328 feed and ingredient samples were collected in six rounds in all 30 districts of Rwanda from March to October 2017. Feed and feed ingredient samples in all four participant categories had high mean levels of total aflatoxin contamination (AFB1 + AFB2 + AFG1 + AFG2), and relatively low mean levels of total fumonisin contamination (FB1 + FB2 + FB3) (Table 1).

### 2.2. Comparison of Feed and Ingredient Sample Contamination over Sample Collection Period

**Figure 1:**
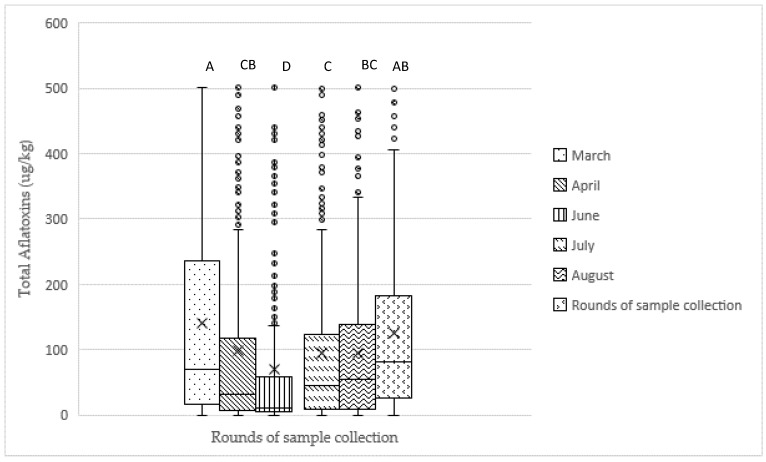
Total aflatoxin contamination (μg/kg) mean, median, range, and outliers by round in feed samples (n = 3328) collected between March and October 2017. “×” and “-“symbols represent the mean and the median in the boxplot, respectively. Levels not connected by the same letter are significantly different (*p* < 0.05).

**Figure 2:**
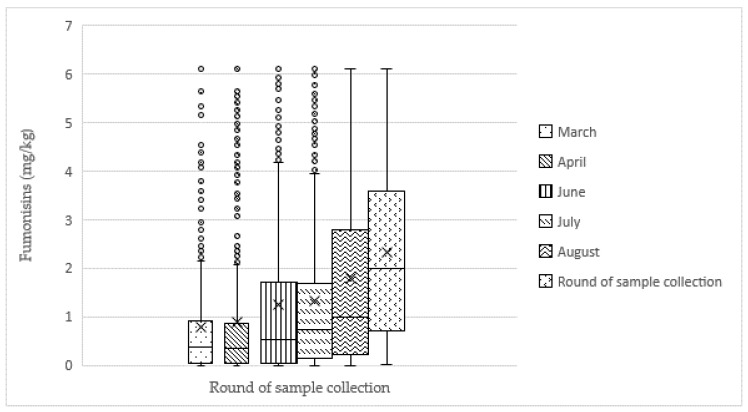
Total fumonisin mean, median, range, and outliers by round in feed samples (n = 3328) collected between March and October 2017. “×” and “-“symbols represent the mean and the median in the boxplot, respectively. There is no statistical difference among different rounds of sample collection (*p* > 0.05).

There was a significant difference in aflatoxin contamination among the six rounds of sample collection (*p* < 0.0001). Samples collected in June (Round 3) had the lowest aflatoxin contamination levels. June corresponds to the maize harvest period, which is characterized by transition from the rainy to the dry season. Aflatoxin contamination level showed a decreasing trend from samples collected in March (Round 1) (mean: 140.78 µg/kg) to samples collected in June (Round 3) (mean: 70.01 µg/kg), then increased from June to October (Round 6) (Figure 1). Fumonisin level showed an increasing trend over the sample collection period. The lowest and highest levels of fumonisin contamination were 0.77 mg/kg and 2.33 mg/kg for Round 1 and Round 6, respectively (Figure 2). However, there is no significant difference among the six rounds of sample collection (*p* > 0.05).

### 2.3. Aflatoxin Contamination Level in Feeds and Feed Ingredients Samples

Complete feeds were the most prevalent samples collected in the survey representing 56.3% of all samples. Complete feeds included feeds from commercial processors, which comprised 4.8% of total samples collected, and feeds mixed by vendors and/or farmers at their own facility or farm, which comprised 51.5% of total samples.

Feed ingredients comprised 43.7% of total samples collected with the most prevalent ingredient, maize bran, comprising 35.9% of total samples. Other ingredient samples collected were rice bran (2.6%), wheat bran (2.4%), brewery byproducts (used only by dairy farmers) (2.3%), and whole maize (0.5%).

**Figure 3:**
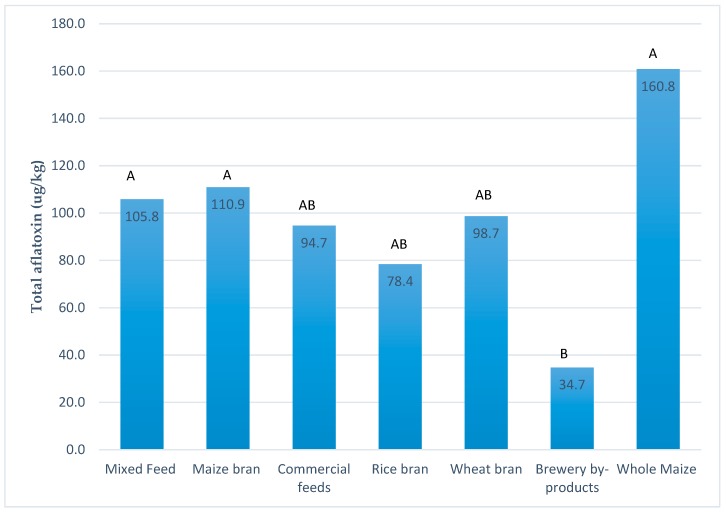
Total aflatoxin means by sample type from most frequent to least frequent feed and feed ingredient type. Levels not connected by the same letter are significantly different (*p* < 0.05).

Aflatoxin contamination by sample type was significantly different (*p* < 0.05). The whole maize samples were most highly contaminated while brewery byproduct samples were least contaminated (Figure 3).

#### Aflatoxin Contamination Weighted Average in Feeds and Feed Ingredients

The weighted average illustrates the relative importance of each feed ingredient or complete feed according to their respective total samples (number of feed type samples/total number of samples × total aflatoxin contamination for feed type), or in other words, an average that results from the multiplication of each feed ingredient or complete feed by a factor indicating its importance. The weighted average for mixed feed and maize bran were 54.5 μg/kg and 39.8 μg/kg, respectively. Other feed sample types had 4.5, 2.1, 2.4, 0.8, and 0.8 μg/kg for commercial feeds, rice bran, wheat bran, brewery byproducts, and whole maize, respectively.

### 2.4. Aflatoxin Contamination of Feed Ingredient Types Throughout Sampling Period

The mean levels of aflatoxins in different feed ingredient types and complete feeds throughout the sampling periods were determined. The low levels for mixed feed, maize bran, rice bran, and wheat bran were 72.4 μg/kg, 74.6 μg/kg, 40.7μg/kg, and 9.9 μg/kg, respectively, and occurred during the June sampling period. The brewery byproduct ingredient had the overall lowest aflatoxin contamination for all sampling periods (Table S2 – Supplement Material).

### 2.5. Aflatoxin Contamination Among Geographical Districts

**Figure 4:**
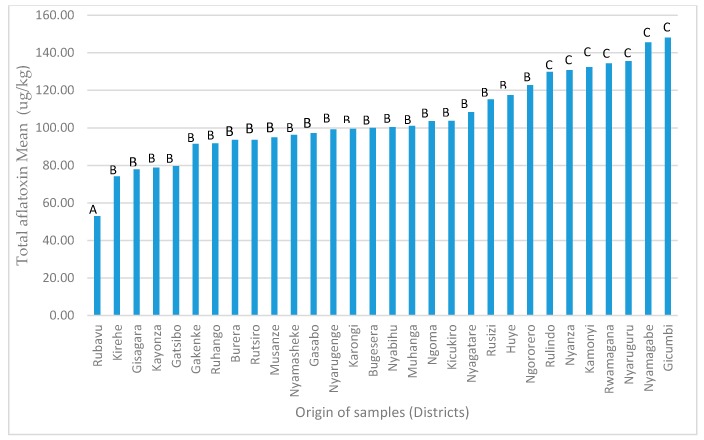
Total aflatoxin means among geographical districts from lowest to highest (different letters indicate statistically significant differences in means with *p* < 0.05).

Location was a significant factor for aflatoxin contamination level. Of the 30 districts in Rwanda, the Rubavu district had the lowest mean aflatoxin level (53.0 μg/kg) and the Gicumbi district had the highest (148.1 μg/kg) (Figure 4).

Maize bran and mixed feed were used across all districts. Rice bran was used only in Gasabo, Huye, Nyamagabe, Kirehe, Rulindo, and Gatsibo districts. Brewery byproduct was used only in Rubavu, Kicukiro, and Musanze districts. Wheat bran was used only in Gisagara, Nyaruguru, Gicumbi, Huye, Nyanza, Nyabihu, Rulindo, and Musanze (Table S3 – Supplement Material).

### 2.6. Socio-Demographic Characteristics of Participants

Socio-demographic characteristics of study participants did not show any statistically significant differences for aflatoxin or fumonisin contamination of the samples collected from their locations (*p* > 0.05). More than 20% of participants were female owners of dairy (8.1%) or poultry farms (12.5%). Additionally, about one-fourth of participants (26%) had a university education. The majority of participants were in the age range of 26–40 years old (56.5%). Moreover, a high proportion of participants, 92.4%, were not aware of aflatoxins, fumonisins, and their adverse effects. Socio-demographic characteristics for feed processors/grain millers were not established, as they have numerous employees in their cooperatives or commercial businesses (Table 2).

### 2.7. Supplement Results

Sample number of each feed ingredient type by each sampling round (Table S4) are provided in Supplementary Materials. For each round, the percentage of feed samples co-contaminated by aflatoxins and fumonisins was calculated (Table S5). The prevalence of percentage of positive samples above the limit of quantification for aflatoxins and fumonisins by sample type and per round of sample was calculated (Table S6).

## 3. Discussion

The current study showed that feed ingredients and complete feeds collected from different participants (dairy farmers, poultry farmers, feed vendors, feed processors) that spanned the feed value chain showed widespread contamination with aflatoxins and fumonisins. The co-occurrence of aflatoxins and fumonisins occurred in 22.9% of feed samples. Co-occurrence of fumonisins and aflatoxins in feeds and feed ingredients has been reported in the East Africa region by several authors. In a report from Tanzania, fumonisin levels in cocontaminated samples ranged from 111 to 11,048 μg/kg (mean = 2157 μg/kg) and aflatoxins from 1 to 151 μg/kg (mean = 44 μg/kg) [1]. Another study in Tanzania reported cocontamination in maize samples [42]. Kang’Ethe et al. reported contamination of aflatoxins and fumonisins in feed samples collected in two Kenyan counties (equivalent to provinces or states) with historical outbreaks of aflatoxin poisoning of people. Up to 56% of feed samples were aflatoxin-positive and 14.6% exceeded the 5 μg/kg limit set by the Food and Agriculture Organization/World Health Organization. The overall average mean was 3.84 μg/kg aflatoxins (range of 0.55 ug/kg to 7.13 ug/kg). Fumonisin B1 was detected in 81.8% of 22 feed samples analyzed [24]. The relatively low aflatoxin contamination levels in these studies compared to our results can be explained, in part, by different interventions to mitigate mycotoxins in maize fields. For instance, the Africa Research in Sustainable Intensification for the Next Generation (Africa RISING/IITA) project intervened in the area of study in Tanzania [42]. Farmers were trained on mitigating mycotoxin contamination along food and feed value chains. In Kenya, different management and mitigation strategies were taken to reduce aflatoxin contamination following the 2005 aflatoxicosis outbreak. Crop rotation, use of certified seeds, use of manure and fertilizers, sorting moldy grains, harvesting at physiological maturity, and proper storage were recommended by policymakers to mitigate aflatoxins and fumonisins at the household level [43]. However, more efforts are still needed to eradicate completely aflatoxin in food and feed value chains.

Fumonisin levels in our study did not exceed European Union (EU) guidance values for feeds for poultry (<4 months) set at 20 mg/kg, nor did fumonisin levels exceed the 50 mg/kg limit for adult ruminants (>4 months) [44]. Likewise, they did not exceed the United States Food and Drug Administration (FDA) guidance levels for maize and maize byproducts intended for animal consumption (i.e., 30 mg/kg total fumonisins for breeding ruminants and breeding poultry) [45]. Aflatoxin contamination of feed vendor samples collected in the current study confirmed a previous study in which 21 feed samples collected in different open markets of Kigali had aflatoxin levels between 100.4 and 168.8 µg/kg [39]. More than 85% of feed ingredients and complete feed samples collected from dairy farms in the current study exceeded the 5 μg/kg AFB1 limit established by the Rwanda Standards Board (RSB) for cattle feed supplements [46]. This standard is the only published standard that addresses the mycotoxin issue in feeds in Rwanda. Aflatoxin contamination in feed ingredients and complete feeds affects animal health and productivity but also endangers public health through their transfer into ASF used as human food, such as the excretion of AFM1 in milk [32].

Aflatoxin contamination showed a significant difference over six sampling periods. Complete feed and feed ingredient samples collected in June had the lowest overall mean of aflatoxin contamination. June corresponds to the maize harvest period, which is characterized by transition from the rainy to the dry season. At harvest, maize is expected to have the lowest level of aflatoxin contamination and, ideally, good postharvest drying and storage practices would prevent molds and associated aflatoxin development. The practical reality is that most maize farmers in Rwanda do not have access to drying and storage technologies that would help them reduce maize to safe storage moisture content quickly and maintain it at safe storage moistures. This lack of technology renders producers unable to take proactive measures to reduce the potential for mold growth and aflatoxin development. Subsequently, maize and maize-derived products have increased risk of aflatoxin accumulation the longer they are stored after harvest. Additionally, millers spray water on maize before hammer milling in order to remove the bran from the kernel more readily. The wetted maize bran is generally accumulated in a pile on the floor of the maize mill that can self-heat and induce further mold growth and aflatoxin development which continues once maize bran is bagged. Therefore, it is not surprising that the current study documented aflatoxin content in feed ingredients and complete feeds that contain maize bran or whole maize increasing the further away sample collection occurred from the June harvest period over the course of this study.

Mbuza et al. reported that maize bran is used as the main basal feedstuff on poultry and broiler farms in Rwanda [40] which was also observed in this study. Maize-based ingredients were either a simple feed ingredient (maize bran) or mixed with other ingredients (mixed feed). The weighted averages were 39.8 μg/kg and 54.5 μg/kg for maize bran and mixed feeds, respectively. Consequently, the more contaminated maize-based ingredients are with aflatoxins, the higher the aflatoxin contamination in feeds. Feed ingredients and complete feeds also showed large standard deviations from the means and many outliers. The high standard deviations are explained by the heterogeneous nature of aflatoxin contamination [47]. The fact that in this study most median values were much greater than mean values suggests the data were positively skewed, with many low values and fewer higher values.

The Rubavu district had the lowest level of aflatoxin contamination, which is likely due to the dominant feed ingredient, brewery byproducts, from a major brewery located there. Bralirwa (from the French acronym “*Bralisseries et Limonaderies du Rwanda*”), the largest brewer and soft beverage company, is located in the Rubavu district and uses mainly barley and other cereals. Brewery byproducts had the lowest aflatoxin levels of any of the sample types collected in this study, likely because of the proactive aflatoxin mitigation measures taken by the brewery (human beverage producer) to minimize aflatoxin contamination in raw ingredients (i.e., barley, rice).

More than 90% of participants in this study reported that they had never heard the words “mycotoxins or aflatoxins” nor their consequences. As a matter of fact, there is no equivalent word for “aflatoxins” in the local Kinyarwanda language. Therefore, an additional question was asked to assess participants’ knowledge of mold contamination in feeds and feed ingredients and their consequences. The vast majority (over 85%) reported to have seen moldy feed ingredients without knowing their consequences. A previous study, targeting maize flour vendors in Kigali’s open markets, reported that all participants (n = 158) were unaware of aflatoxins [39]. Nyangi et al. reported that 62% of farmers in the Babati district of Tanzania were aware of mycotoxins and their consequences thanks to the intervention of Africa RISING/IITA and NGOs [42]. Raising awareness of aflatoxins and their consequences has to be considered as a key element in any aflatoxin mitigation strategy. Implementation will not succeed if farmers do not first understand the danger of aflatoxins.

Absence of appropriate regulations contribute to the mycotoxin threat in Rwanda. All participants in this study were unaware of the one existing standard for aflatoxins in dairy feed supplements. In the African context, establishment of standards and enforcement of regulations is currently driven by trade and the desire to comply with export regulations [48]. The inability to enforce even the single, existing aflatoxin standard renders it useless and gives the false impression of controlling the situation [49]. Sirma et al. suggested that aflatoxin standards for food and feeds would be more effective if the context of local conditions were considered, such as typical crops used as ingredients, capacity to enforce regulations, differentiated aflatoxin contamination levels in food and feeds, regional standards, and societal concerns (food and nutrition security) [50].

Results of this study will inform current regional and continental efforts to leverage aflatoxin mitigation strategies. Recently, the East African Community (EAC), a regional intergovernmental organization, provided options for disposal and alternative uses of aflatoxin-contaminated commodities [51]. Moreover, ten years ago, the African Union established the Partnership for Aflatoxin Control in Africa (PACA). Its role is to provide leadership, and coordinate and increase effective aflatoxin control in Africa [52]. This study will contribute to PACA’s resources and knowledge.

## 4. Conclusions

This study quantified aflatoxin and fumonisin contamination in the feed value chain of Rwanda over a seven-month period in 2017. Based on the findings, further studies are recommended to explore existing strategies for mitigating mycotoxins in Rwanda’s feed value chain. Interventions are recommended to focus on early value-chain participants such as maize farmers because end-users such as dairy farmers are subject to what is available from feed suppliers. Standards and regulations adapted to the context of Rwanda are needed because more than 85% of feed ingredients and complete feed samples collected from dairy farms in the current study exceeded the 5 μg/kg AFB1 limit established by the Rwanda Standards Board (RSB) for cattle feed supplements. An awareness campaign should be initiated to improve feed and ASF safety for the benefit of dairy and poultry farmers and consumers because more than 90% of study participants were unaware of aflatoxins and fumonisins, and their consequences.

The specific conclusions of this study are:

- Feed ingredients and complete feeds were found to be contaminated with aflatoxins and fumonisins. Dairy farmers, poultry farmers, feed vendors, and feed processors had mean aflatoxin levels of 108.83 µg/kg (Median (MD): 43.65 µg/kg), 103.81µg/kg (MD: 48.4 µg/kg), 88.64 µg/kg (MD: 30.90 µg/kg), and 94.95 µg/kg (MD: 70.45 µg/kg), respectively, which were above EU and FDA limits for dairy and poultry. However, fumonisins did not exceed the EU and FDA guidance values for feeds for mature poultry set at 20 mg/kg, and 30 mg/kg for breeding ruminants and breeding poultry.
- Considering the weighted means, mixed feed (54.5 μg/kg) and maize bran (39.8 μg/kg) were the two major contributors to aflatoxin contamination.
- Two risk factors, district and sampling period, showed a significant effect (*p* < 0.05) on aflatoxin contamination of feed ingredients and complete feeds.
- None of the study participants were aware of the existence of the RSB standard for AFB1 in cattle feed supplements published in Rwanda.

## 5. Materials and Methods

### 5.1. Study Areas and Identification of Participants

Six rounds of sample collection were carried out in all 30 districts of Rwanda between March and October 2017. Four categories of participants were recruited for this study: feed processors (including finished feeds, and maize mills selling maize bran), feed vendors, dairy farmers, and poultry farmers. A comprehensive list of eligible participants was obtained from the District Veterinary Officer (DVO) of each of the 30 districts. Recruitment targets for the study were 20 participants per district. When the number of potential participants per district was less than 20, all participants were included. For districts with more than 20 potential participants, a minimum of 20 participants were selected randomly. In the local Rwandan context, an individual with even one cow is considered a dairy farmer. There are a large number of farmers, particularly in rural areas, who rely on grazing and do not use animal feeds. To avoid such bias in the current study, we set two inclusion criteria prior to recruiting participants in the category of dairy farmers: they must have at least two cows and use feeds as the principal ration (or at least as a dietary supplement). Poultry farmers considered for participation in the study were broiler and layer farmers who sought to generate income from their products. A total of 612 participants were included consisting of 10 feed processors/maize millers, 68 feed vendors, 225 dairy farmers, and 309 poultry farmers. Due to their relatively small number, all identified feed processors/maize millers were included.

### 5.2. Questionnaire Development and Administration

Structured questionnaires were designed to obtain socio-demographic information, participant knowledge and awareness of mycotoxins, and general feeding practices (i.e., ingredients used, sourcing and storage of ingredients or complete feeds). Questionnaires were specific to each of the four participant categories and were administered to participants by enumerators during the first round of sample collection.

### 5.3. Sample Collection

From each participant, a sample (approximately 2 kg) of feed or feed ingredients (mixed feed, commercial feed, rice bran, wheat bran, or maize bran, breweryby products, and whole maize) was collected. Samples were collected from the same participants for each of the six rounds of the study. If samples were not available from some participants in a round, they were recorded as missing samples. Samples were stored in a freezer (–20 °C) as soon as possible after collection to halt fungal growth and additional aflatoxin and fumonisin accumulation.

### 5.4. Sample Preparation

After removal from the freezer, complete feed and feed ingredient samples were allowed to equilibrate to room temperature before being ground in their entirety using a Romer Series II® subsampling mill (Romer Labs, Inc., Union, MO, USA). A minimum of 200 g ground subsamples were kept and, again, stored in the freezer (–20 °C) prior to analysis. To prevent cross-contamination, the mill was cleaned thoroughly between samples using a vacuum (according to manufacturer recommendation) and approximately the first 100 g of every ground sample exiting the mill was discarded. At least once per day, the mill was disassembled and an intensified dry cleaning was performed.

### 5.5. Sample Extraction

A deviation from the Helica Total Aflatoxin Assay extraction protocol was made in consultation with the manufacturer to harmonize the extraction process for both mycotoxins. Our working procedure was as follows. A 20 g ground test portion was weighed from each subsample, mixed with 40 ml 90% methanol (ACS grade, Finar Ltd., Gujarat, India), and shaken in a sealed container for a minimum of two minutes. Particulate matter was allowed to settle; then, 5 to 10 mL of extract was filtered through Whatman #1 filter paper. The resultant filtrate was collected and the pH was adjusted to 7.0 (+ 1.0), with 25% NaOH or 2 M HCl. Approximately half of the collected filtrate was stored and refrigerated (5 °C) in a labeled centrifuge tube to be used in the fumonisin ELISA assay. From the remaining collected filtrate, 1 ml was diluted in 1.5 ml 57% methanol in a centrifuge tube to bring the final concentration to 70% methanol, which is prescribed for the total aflatoxin ELISA assay. If samples contained aflatoxin or fumonisin levels exceeding the operating range of the assay, additional dilutions were made using 70% methanol. All 90% and 70% methanol extracts were stored in the refrigerator prior to laboratory analyses. Filtered extracts were not used after one week in the refrigerator due to loss of aflatoxin stability past this time.

### 5.6. Sample Analysis

All collected feed and feed ingredient samples were analyzed using competitive Total Aflatoxin (AFB1 + AFB2 + AFG1 + AFG2) Enzyme-Linked Immunosorbent Assay (ELISA) (Catalog #941AFL01M-96) and Fumonisin (FB1 + FB2 + FB3) Assay (Catalog #951FUMO01C-96) (Helica Biosystems, Santa Ana, CA, USA) according to manufacturer specifications. For total aflatoxin, 200 μL of the aflatoxin-HRP conjugate was dispensed into each 96-well mixing plate and 100 μL of either standard or sample was added to the appropriate mixing well containing conjugate. Wells were mixed by pipetting up and down at least three times. Content from each mixing well (100 μL) was transferred to a corresponding antibody-coated microtiter well and incubated for 15 min. PBS-Tween wash buffer was used to rinse the plate wells for five washes. A substrate reagent (100 μL) was added to each antibody-coated microtiter well and incubated for 5 min. A stop solution (100 μL) was added in the same sequence and at the same place as where the substrate reagent was added. The optical density (OD) was read for each well of the plate using a plate reader with a 450 nm filter (Thermo Scientific Multiskan FC, Thermo Fisher Scientific, Ratastie, Finland). For fumonisin kits, the assay procedure was the same as for the total aflatoxin assay procedure, with the following exceptions: 100 μL each of two conjugate solutions (A and B) were added to each well of the 96-well mixing plate, and incubation times were 10 min after transfer to the corresponding antibody-coated 96-well plate and 10 min after adding the substrate reagent. The OD readings generated by the standard solutions on each 96-well plate were used to generate a logit regression equation for that plate, which was subsequently used to calculate the total aflatoxin and fumonisin concentrations in sample extracts on the corresponding plate. The final concentration was adjusted according to the dilution factor. The operating range was 5–500 µg/kg and 1–6 mg/kg for total aflatoxins and fumonisins, respectively.

### 5.7. External Validation

Subsets of feed and ingredient samples that represented the varying types of matrices that were anticipated to be collected during our survey were obtained prior to the first round of sample collection. These samples were sent to the Bioscience eastern and central Africa—International Livestock Research Institute (BecA-ILRI) in Nairobi, Kenya, for performance validation of ELISA assays relative to high-performance liquid chromatography (HPLC) and fluorometry methods (VICAM Aflatest®, Watertown, ME, USA). All methods tested had an excellent correlation (*r*^2^ = 0.95). Based on these results, the ELISA method from Helica Biosystems (Santa Ana, CA, USA) was selected because it performed best on the most prevalent matrices.

### 5.8. Internal Validation

For each ELISA 96-well plate, six ready-to-use standards were provided to establish a calibration curve ranging from 0.0 to 4.0 ng/mL for the total aflatoxin ELISA kit and from 0.0 to 150.0 ng/mL for the fumonisin ELISA kit. Spike recoveries and the coefficient of variation (CV) were calculated for each aflatoxin (0, 0.2, 0.5, 1, 2, and 4 ng/mL) and fumonisin (0, 2.5, 7.5, 20, 50, and 150 ng/mL) standard concentrations used. The acceptable level of CV was set at 5%. The regression coefficient (*r*^2^) was calculated for calibration curve linearity for each plate. The minimum acceptable level for *r*^2^ was set at 0.98. Aflatoxin and fumonisin quality control materials (QCM) (Biopure®, Romer Labs, Inc., Tulln, Austria) were included on select ELISA plates throughout the analysis period to monitor accuracy. QCMs were ground corn contaminated with aflatoxins (aflatoxin B1: 8.8 ± 3.1 μg/kg, aflatoxin B2: <1 μg/kg, aflatoxin G1: <1 μg/kg, and aflatoxin G2:1 μg/kg) and fumonisins (fumonisin B1: 1232 ± 152 μg/kg, fumonisin B2: 282 ± 42 μg/kg, fumonisin B3: 139 + 29 μg/kg). The spike recovery for aflatoxins was 89% and 97% for fumonisins.

### 5.9. Statistical Analysis

A fixed effect model (SAS 9.4) was used to calculate the association between risk factors and the level of aflatoxins and fumonisins in feed samples using Analysis of Variance (ANOVA). Aflatoxin levels were log transformed due to the skewedness distribution. Gender, education, and age of the participants, origin of the sample (district), mycotoxin awareness, and sample collection round were fitted as fixed effect variables in the model. To calculate different descriptive statistic parameters, for samples lower than the limit of quantification of the test kit for total aflatoxins or fumonisins, the data point was replaced by a value equal to half of the limit of quantification (2.5 µg/kg and 0.05 mg/kg for total aflatoxins and fumonisins, respectively) to avoid biasing the results. Samples exceeding the operating range (500 µg/kg and 6 mg/kg for total aflatoxins and fumonisins, respectively) were replaced by the values of 501 µg/kg and 6.1 mg/kg. Thus, absolute maximum values could not be determined. However, only 4.3% of total samples exceeded the maximum operating limit.

### 5.10. Research Ethics

In compliance with institutional ethics requirements, University of Rwanda (004/DRIPGS/2017) and Iowa State University (IRB ID: 17-083), participants gave their informed consent for inclusion before they participated in the study. Before signing the consent form, participants received an explanation of the objectives and anticipated outcomes of the project.

## Figures and Tables

**Table 1:** Aflatoxin and fumonisin mean, standard deviation (SD), and median values in feed and feed ingredient samples among different participant categories.

		Aflatoxins (µg/kg)			Fumonisins (mg/kg)		
	Sample Number (%)	Mean	SD	Median	Mean	SD	Median
***Dairy Farmers***	1180 (35.46)	108.83	144.90	43.65	1.52	1.83	0.71
***Poultry Farmers***	1726 (51.86)	103.81	135.91	48.40	1.21	1.54	0.56
***Feed Vendors***	365 (10.97)	88.64	128.59	30.90	1.48	1.74	0.76
***Feed Processors***	57 (1.71)	94.95	103.19	70.45	1.03	1.27	0.47

Among different participants, mixed feed and maize had lowest contamination during the June sampling period in dairy farmers (59.3 μg/kg and 86.6 μg/kg, respectively) and poultry farmers (74.6 μg/kg and 56.9 μg/kg, respectively) (Table S1 – Supplement Material). Statistical analysis did not show that participant category was a significant factor for aflatoxin and fumonisin contamination (*p* < 0.05).

**Table 2:** Socio-demographic characteristics of study participants.

Factors		Dairy Farmers		Poultry Farmers		Feed Vendors		F. Pr./G. M.^a^		Overall	
		#	%	#	%	#	%	#	%	#	%
**Gender**	Male	176	29.2	234	38.9	50	8.30	-	-	460	76.4
	Female	49	8.10	75	12.5	18	3.00	-	-	142	23.6
**Education**	Primary	88	14.6	114	18.9	13	2.20	-	-	215	35.7
	Secondary	74	12.3	98	16.3	36	6.00	-	-	208	34.6
	University	53	8.80	87	14.5	19	3.20	-	-	159	26.4
	None	9	1.50	9	1.50	0	0.00	-	-	18	2.99
	Other	1	0.200	1	0.200	0	0.00	-	-	2	0.330
**Age**	18–25	0	0	8	2.59	13	19.1	-	-	21	3.49
	26–40	108	48.0	193	62.5	39	57.4	-	-	340	56.5
	>40	117	52.0	108	35.0	16	23.5	-	-	241	40.0
**Awareness**	Yes	8	1.30	27	4.50	9	1.50	2	0.300	46	7.62
	No	213	35.3	278	46.0	59	9.80	8	1.30	558	92.4

#: Total number of participants in each category per each socio-demographic characteristic considered; %: Percentage of participants in each category per each socio-demographic characteristic considered; ^a^ Feed Processors/Grain Millers were cooperatives or commercial companies. Several factors of their employees (i.e., gender, education, and age) were not recorded.

## Supplementary Materials

The following are available online at https://www.mdpi.com/2072-6651/11/5/270/s1, Table S1: Comparison of aflatoxin levels (means in μg/kg) in feed ingredients and mixed feed in different categories of study participants, Table S2: Total mean aflatoxin levels of feed ingredient types over sampling periods, Table S3: Total mean aflatoxin levels (μg/kg) for ingredient types and sampling periods across geographical districts, Table S4: The sample number of each feed ingredient type by each sampling round, Table S5: The co-occurrence of aflatoxins and fumonisins in the samples, Table S6: Prevalence (percentage of positive samples) above the limit of quantification for aflatoxins and fumonisins by sample type and per round of sample.
