# Supplementary material for: Assessment of Aflatoxin and Fumonisin Contamination and Associated Risk Factors in Feed and Feed Ingredients in Rwanda
Source: Toxins (Basel). 2019 May 14;11(5):270. doi: 10.3390/toxins11050270 (PMC6563260; doi:10.3390/toxins11050270)
Supplement: Supplementary file 1 [file toxins-11-00270-s001.pdf]

Supplementary Materials: Assessment of Aflatoxin and Fumonisin Contamination and Associated Risk Factors in Feed and Feed Ingredients in Rwanda

Kizito Nishimwe, Erin Bowers, Jean de Dieu Ayabagabo, Richard Habimana, Samuel Mutiga and Dirk Maier

Table S1. Comparison of aflatoxin levels (means in µg/kg) in feed ingredients and mixed feed in different categories of study participants.

Participants	Sampling Time	Mixed Feed (µg/kg)	Maize Bran (µg/kg)	Commercial Feeds (µg/kg)	Rice Bran (µg/kg)	Wheat Bran (µg/kg)	Brewery By-products (µg/kg)	Whole Maize (µg/kg)
**Dairy Farmers**	March	178.8	157.6	-	57.1	25.6	7.6	22.7
	April	128.8	111.0	-	98.8	119.6	2.8	70.1
	June	59.3	86.6	-	48.9	6.5	4.0	169.8
	July	119.5	91.0	-	179.4	111.7	26.9	3.2
	August	132.5	119.8	-	109.8	79.4	24.5	3.6
	October	123.6	159.4	-	148.0	121.6	43.0	154.0
**Poultry Farmers**	March	146.5	146.8	-	34.9	94.8	-	247.3
	April	108.4	80.9	-	22.3	136.1	-	37.0
	June	74.6	56.9	-	22.3	22.3	-	20.4
	July	103.5	101.1	-	65.5	3.0	-	52.8
	August	92.3	90.8	-	12.0	165.5	-	24.4
	October	112.9	116.8	-	103.5	63.9	-	63.9
**Feed Vendors**	March	-	148.5	94.3	-	-	-	-
	April	-	53.7	53.7	-	-	-	-
	June	-	72.5	77.7	-	-	-	-
	July	-	59.1	82.1	-	-	-	-
	August	-	118.9	95.1	-	-	-	-
	October	-	99.6	94.4	-	-	-	-
**Feed Processors**	March	-	148.6	239.6	-	-	-	-
	April	-	121.5	123.0	-	-	-	-
	June	-	12.5	77.3	-	-	-	-

	July	-	101.0	70.3	-	-	-	-
	August	-	80.8	35.1	-	-	-	-
	October	-	99.6	94.4	-	-	-	-

Table S2. Total mean aflatoxin levels of feed ingredient types over sampling periods.

Sampling Time	Mixed Feed (μg/kg)	Maize Bran (μg/kg)	Commercial feeds(μg/kg)	Rice bran(μg/kg)	Wheat bran(μg/kg)	Brewery by-products(μg/kg)	Whole Maize (μg/kg)
**March**	151.4	149.0	105.5	50.3	37.8	7.6	157.5
**April**	111.5	93.7	67.5	79.6	122.5	2.8	45.2
**June**	72.4	74.6	77.7	40.7	9.9	4.0	70.2
**July**	105.8	85.8	81.1	141.5	96.2	26.9	1.8
**August**	98.1	113.2	90.8	79.7	92.6	24.5	18.5
**October**	114.5	147.6	94.3	131.8	110.1	43.0	110.9

Table S3. Total mean aflatoxin levels (μg/kg) for ingredient types and sampling periods across geographical districts.

District	Sampling Time	Mix	Maize Bran	Commercial Feeds	Rice Bran	Wheat Bran	Brewery By-products	Whole Maize
**Gasabo**	March	93.3	154.5	-	22.3	-	-	-
	April	123.5	118.3	-	28.2	-	-	-
	June	116.1	36.4	-	104.6	-	-	-
	July	43.8	82.4	-	198.4	-	-	-
	August	86.9	108.5	-	48.3	-	-	-
	October	62.2	169.2	-	20.3	-	-	-
**Kayonza**	March	92.3	95.1	-	-	-	-	-
	April	220.9	111.0	-	-	-	-	-
	June	75.8	51.2	-	-	-	-	-
	July	98.9	53.2	-	-	-	-	-
	August	22.3	38.2	-	-	-	-	-
	October	-	85.9	-	-	-	-	-
**Burera**	March	168.9	184.4	-	-	-	-	247.1
	April	90.7	13.8	-	-	-	-	6.4

	June	30.8	7.7	-	-	-	-	8.6
	July	112.6	117.1	-	-	-	-	10.5
	August	108.6	150.4	-	-	-	-	0.3
	October	92.2	82.2	-	-	-	-	59.1
**Rutsiro**	March	172.1	0.3	-	-	-	-	-
	April	165.6	8.3	-	-	-	-	-
	June	8.4	0.3	-	-	-	-	-
	July	201.9	123.5	-	-	-	-	-
	August	66.2	66.5	-	-	-	-	-
	October	34.0	68.7	-	-	-	-	-
**Gisagara**	March	0.3	20.0	-	-	8.6	-	-
	April	7.0	12.2	-	-	13.7	-	-
	June	64.9	501.0	-	-	0.3	-	-
	July	7.4	0.3	-	-	321.4	-	-
	August	0.3	-	-	-	53.1	-	-
	October	-	158.4	-	-	40.8	-	-
**Nyaruguru**	March	250.8	168.1	-	-	41.9	-	-
	April	129.7	81.7	-	-	157.4	-	-
	June	223.6	40.2	-	-	0.3	-	-
	July	154.7	91.4	-	-	371.4	-	-
	August	128.9	198.5	-	-	159.6	-	-
	October	104.6	124.3	-	-	-	-	-
**Gicumbi**	March	153.4	218.9	11.7	-	30.4	-	-
	April	136.9	26.5	50.3	-	220.0	-	-
	June	118.2	85.5	55.2	-	0.3	-	-
	July	138.8	124.6	234.5	-	16.8	-	-
	August	146.5	240.5	138.6	-	149.3	-	-
	October	192.8	273.5	42.1	-	304.9	-	-
**Huye**	March	167.4	255.4	266.8	22.7	20.6	-	-
	April	113.2	80.2	367.5	15.9	116.3	-	-

	June	70.6	109.5	#DIV/0!	46.7	2.4	-	-
	July	117.5	73.3	79.3	133.7	93.3	-	-
	August	163.5	58.8	0.3	15.5	83.5	-	-
	October	158.7	124.4	115.1	199.9	93.8	-	-
**Gakenke**	March	177.3	102.6	13.8	-	-	-	-
	April	52.0	49.6	0.3	-	-	-	-
	June	102.4	171.8	15.6	-	-	-	-
**Gakenke**	July	101.9	70.5	41.0	-	-	-	-
	August	107.7	161.5	104.9	-	-	-	-
	October	61.2	56.2	0.3	-	-	-	-
	March	186.9	202.0	-	-	-	-	-
	April	98.5	88.8	-	-	-	-	-
	June	75.7	58.7	-	-	-	-	-
**Kamonyi**	July	117.1	32.6	-	-	-	-	-
	August	79.5	199.7	-	-	-	-	-
	October	256.4	17.9	-	-	-	-	-
	March	287.4	220.2	-	0.3	-	-	-
	April	170.7	47.0	-	57.7	-	-	-
**Nyamagabe**	June	214.7	90.5	-	17.5	-	-	-
	July	73.0	41.1	-	97.5	-	-	-
	August	126.2	159.5	-	253.3	-	-	-
	October	194.9	140.6	-	9.5	-	-	-
	March	128.2	208.9	-	-	-	-	-
	April	92.4	131.4	-	-	-	-	-
**Ngoma**	June	32.8	8.9	-	-	-	-	-
	July	97.3	110.6	-	-	-	-	-
	August	106.7	71.6	-	-	-	-	-
	October	92.8	159.0	-	-	-	-	-
**Kirehe**	March	130.9	180.5	-	14.8	-	-	34.3
	April	35.5	54.6	-	29.6	-	-	35.6

	June	17.6	9.9	-	0.3	-	-	18.0
	July	126.4	86.8	-	41.5	-	-	61.8
	August	72.4	3.1	-	0.3	-	-	76.3
	October	87.6	332.3	-	137.9	-	-	130.7
**Ngororero**	March	181.8	139.8	0.3	-	-	-	-
	April	247.7	152.8	159.1	-	-	-	-
	June	73.7	118.2	82.6	-	-	-	-
	July	103.2	1.5	153.5	-	-	-	-
	August	103.2	90.2	120.4	-	-	-	-
	October	68.1	199.8	52.2	-	-	-	-
**Karongi**	March	64.3	230.3	-	-	-	-	-
	April	61.4	98.6	-	-	-	-	-
	June	89.2	66.8	-	-	-	-	-
	July	54.2	157.2	-	-	-	-	-
	August	67.8	48.2	-	-	-	-	-
	October	99.6	183.8	-	-	-	-	-
**Nyagatare**	March	51.2	129.8	-	-	-	-	501.0
	April	88.1	93.9	-	-	-	-	84.7
	June	5.4	96.7	-	-	-	-	0.3
	July	91.2	42.5	-	-	-	-	145.4
	August	112.5	150.4	-	-	-	-	-
	October	93.2	178.5	-	-	-	-	-
**Nyanza**	March	154.5	88.5	-	-	94.8	-	-
	April	225.7	98.1	-	-	73.6	-	-
	June	74.1	106.9	-	-	52.8	-	-
	July	149.4	55.0	-	-	0.3	-	-
	August	293.4	83.6	-	-	254.7	-	-
	October	181.0	149.0	-	-	20.1	-	-
**Ruhango**	March	135.9	132.9	-	-	-	-	139.2
	April	49.9	501.0	-	-	-	-	7.5

	June	41.0	75.5	-	-	-	-	0.3
	July	113.0	136.7	-	-	-	-	44.8
	August	86.8	44.2	-	-	-	-	31.1
	October	63.6	154.5	-	-	-	-	185.3
**Kicukiro**	March	153.9	120.0	-	-	20.4	0.3	-
	April	203.4	114.6	-	-	0.3	0.3	-
	June	64.2	96.5	-	-	12.4	-	-
	July	125.9	81.3	-	-	-	198.5	-
	August	92.6	120.1	-	-	0.3	7.6	-
	October	70.1	67.7	-	-	9.4	328.1	-
**Muhanga**	March	180.2	175.0	-	-	-	-	-
	April	103.4	68.2	-	-	-	-	-
	June	50.4	101.6	-	-	-	-	-
	July	35.5	151.8	-	-	-	-	-
	August	89.2	104.4	-	-	-	-	-
	October	30.2	103.4	-	-	-	-	-
**Rwamagana**	March	277.0	278.1	452.5	-	101.0	-	-
	April	165.2	108.1	8.8	-	68.4	-	-
	June	50.4	101.6	-	-	-	-	-
	July	60.5	163.9	24.4	-	-	-	-
	August	53.4	87.7	14.5	-	-	-	-
	October	96.0	231.3	34.2	-	-	-	-
**Nyabihu**	March	106.4	288.8	-	-	88.6	-	-
	April	49.9	266.5	-	-	266.4	-	-
	June	34.5	254.5	-	-	5.4	-	-
	July	101.1	246.6	-	-	5.7	-	-
	August	44.5	47.1	-	-	76.2	-	-
	October	188.1	313.2	-	-	107.7	-	-
**Rulindo**	March	152.9	58.5	-	170.4	29.9	-	13.3
	April	105.7	78.8	-	236.6	144.6	-	140.0

	June	126.8	139.5	-	5.8	12.1	-	501.0
	July	79.5	148.5	-	188.9	78.2	-	6.2
	August	158.5	100.6	-	89.1	55.5	-	6.9
	October	185.8	175.2	-	298.1	331.2	-	-
**Gatsibo**	March	51.6	80.3	-	0.3	-	-	-
	April	142.0	137.2	-	0.3	-	-	-
	June	26.0	42.7	-	11.6	-	-	-
	July	52.8	88.3	-	124.0	-	-	-
	August	43.6	70.4	-	53.1	-	-	-
	October	71.9	99.4	-	-	-	-	-
**Nyarugenge**	March	123.8	110.4	24.3	-	-	-	324.2
	April	101.5	74.1	18.6	-	-	-	81.2
	June	34.3	65.6	32.0	-	-	-	68.7
	July	121.2	84.3	39.3	-	-	-	43.7
	August	118.7	208.2	130.7	-	-	-	14.1
	October	37.9	148.0	73.9	-	-	-	41.2
**Rusizi**	March	152.1	167.1	-	-	-	-	-
	April	120.4	173.8	-	-	-	-	-
	June	44.8	96.5	-	-	-	-	-
	July	100.8	72.0	-	-	-	-	-
	August	46.4	62.4	-	-	-	-	-
	October	168.3	217.1	-	-	-	-	-
**Rubavu**	March	84.2	72.1	48.6	-	-	4.2	-
	April	14.4	9.7	9.8	-	-	5.3	-
	June	59.8	13.0	6.0	-	-	0.3	-
	July	135.5	27.0	99.2	-	-	0.3	-
	August	46.2	31.4	33.4	-	-	50.3	-
	October	109.5	89.5	456.9	-	-	20.0	-
**Nyamasheke**	March	128.5	39.3	349.4	-	-	-	-
	April	64.2	57.1	0.3	-	-	-	-

	June	57.5	160.3	501.0	-	-	-	-
	July	154.0	46.1	0.3	-	-	-	-
	August	132.9	57.8	11.9	-	-	-	-
	October	118.3	34.3	0.3	-	-	-	-
**Musanze**	March	248.2	165.7	239.6	-	-	14.7	-
	April	171.6	47.5	123.0	-	-	0.3	-
	June	86.4	50.7	77.3	-	-	15.3	-
	July	90.8	58.9	70.3	-	-	24.0	-
	August	116.6	97.5	35.1	-	-	0.3	-
	October	118.9	107.5	94.4	-	-	6.2	-
**Bugesera**	March	160.1	70.1	6.9	-	-	-	-
	April	102.9	110.9	10.7	-	-	-	-
	June	40.8	131.2	-	-	-	-	-
	July	172.0	18.9	-	-	-	-	-
	August	36.4	156.8	195.3	-	-	-	-
	October	56.4	136.3	-	-	-	-	-

Table S4. The sample number of each feed ingredient type by each sampling round.

	**Round 1**	**Round 2**	**Round 3**	**Round 4**	**Round 5**	**Round 6**	**Total**
**Maize Bran**	248	223	177	198	210	135	1191
**Mixed Feed**	298	303	331	305	240	247	1724
**Rice Bran**	13	13	13	15	19	15	88
**Whole Maize**	10	3	1	0	3	0	17
**Commercial Feeds**	13	26	23	28	34	28	152
**Brewery By-products**	13	14	10	12	16	12	77
**Wheat Bran**	17	11	15	11	17	8	79
**Total**	612	593	570	569	539	445	3328

Table S5. The co-occurrence of aflatoxins and fumonisins in the samples.

	**Round 1**	**Round 2**	**Round 3**	**Round 4**	**Round 5**	**Round 6**	**Total**
**Number of Samples**	131	93	355	69	69	45	762
**Percentage**	21.4	15.7	62.9	12.1	12.8	10.1	22.9

Table S6. Prevalence (percentage of positive samples) above the limit of quantification for aflatoxins and fumonisins by sample type and per round of sample.

	Round 1 (%)		Round 2 (%)		Round 3 (%)		Round 4 (%)		Round 5 (%)		Round 6 (%)		Total (%)	
	**Aflatoxins**	**Fumonisins**	**Aflatoxins**	**Fumonisins**	**Aflatoxins**	**Fumonisins**	**Aflatoxins**	**Fumonisins**	**Aflatoxins**	**Fumonisins**	**Aflatoxins**	**Fumonisins**	**Aflatoxins**	**Fumonisins**
**Maize Bran**	89.9	29	79.8	28.6	77.4	42.4	84.9	44.9	79	48.8	95.6	67.4	84.4	43.4
**Mixed Feed**	90.6	21.8	81.5	22.4	77.6	39.3	88.5	42	84.2	45.4	94.7	63.6	86.2	39.1
**Rice Bran**	61.5	0	53.9	7.7	53.9	38.5	73.3	26.7	73.7	52.6	80	66.7	66	32
**Whole Maize**	90	100	66.7	0	100	0	0	0	66.7	33.3	0	0	53.9	22
**Commercial Feeds**	76	7.7	80.8	3.9	82.6	43.5	89.3	46.4	97.1	64.7	82.1	82.1	84.8	41
**Brewery By-products**	23.1	100	14.3	14.3	10	100	16.7	16.7	50	56.3	75	58.3	31.5	57.6
**Wheat Bran**	64.7	11.8	72.7	27.3	60	100	100	36.4	100	53	100	62.5	82.9	48.5
**Total**	71	38.6	64.2	14.8	65.9	51.9	64.7	30.4	78.7	51	75.4	57.2	69.9	40.6
